# Selection of Optimal Candidates for Cytoreductive Nephrectomy in Patients with Metastatic Clear Cell Renal Cell Carcinoma: A Predictive Model Based on SEER Database

**DOI:** 10.3389/fonc.2022.814512

**Published:** 2022-01-21

**Authors:** Yishan Zhang, Jintao Hu, Jingtian Yang, Yingwei Xie, Zhiliang Chen, Wentai Shangguan, Jinli Han, Wang He, Jingyin Yang, Zaosong Zheng, Qiyu Zhong, Dingjun Zhu, Wenlian Xie

**Affiliations:** ^1^ Department of Urology, Sun Yat-sen Memorial Hospital, Sun Yat-sen University, Guangzhou, China; ^2^ Guangdong Provincial Key Laboratory of Malignant Tumor Epigenetics and Gene Regulation, Sun Yat-sen Memorial Hospital, Sun Yat-sen University, Guangzhou, China; ^3^ Guangdong Clinical Research Center for Urological Diseases, Guangzhou, China

**Keywords:** cytoreductive nephrectomy, metastatic renal cell carcinoma, clear cell renal cell carcinoma, nomogram, SEER database

## Abstract

**Background:**

Currently, the progress of targeted drugs in the treatment of metastatic clear cell renal cell carcinoma (mccRCC) is limited. Cytoreductive nephrectomy (CN), as an alternative treatment, can improve the prognosis of patients with metastatic renal cell carcinoma to some extent. However, it is unclear which patients would benefit from this tumor reduction operation. As a consequence, we developed a predictive model to identify patients who may well benefit from CN in terms of survival.

**Methods:**

We identified patients with metastatic clear cell renal cell carcinoma retrospectively from the Surveillance, Epidemiology, and End Results (SEER) database (2010–2015) and classified them into surgery and non-surgery groups. Propensity score matching (PSM) was performed to balance the baseline characteristics. Patients who survived longer than the median overall survival (OS) of no-surgery group were defined as surgical-benefit patients. Then, we developed a predictive model based on preoperative characteristics using multivariable Logistic regression. Calibration curves and the area under the receiver operating characteristic (AUC) were used to evaluate the efficiency of the predictive model. The clinical value of the nomogram was assessed utilizing decision curve analysis (DCA).

**Results:**

Our study collected 5544 patients from the SEER database, with 2352(42.4%) receiving cytoreductive surgery. Overall survival (OS) was longer in the CN group than in the non-surgery group after 1:1 propensity scoring matching (median OS: 19 months vs 7 months; hazard ratio (HR) =0.4106, P< 0.001). In the matched surgery group, 65.7% (367) patients survived more than 7 months after the operation and they were considered to benefit from CN. The predictive model performed well on both the training group (AUC=73.4%) and the validation group (AUC=71.9%) and the calibration curves indicated a high degree of consistency. The decision curve analysis curve demonstrated the clinical utility. We classified surgical patients into the beneficial group and non-beneficial group by using the predictive model, then discovered a substantial difference in OS between the two groups.

**Conclusions:**

We developed a nomogram to select ideal mccRCC patients who might benefit from cytoreductive nephrectomy. Clinicians could make a more precise treatment strategy for mccRCC patients.

## Introduction

Renal cell carcinoma (RCC) is one of the most prevalent types of malignant tumor in the urinary system, accounting for 3% of all malignancies globally. RCC is divided pathologically into 3 main types: clear cell renal cell carcinoma (ccRCC), papillary renal cell carcinoma (pRCC), and chromophobe renal cell carcinoma (chRCC). Among these types, ccRCC is the most prevalent histological subtype (about 80%) and has a poorer prognosis than pRCC and chRCC (1, 2). Although more and more tiny renal masses are detected by various imaging tests, there are 17% of RCC patients diagnosed with metastatic disease (3, 4).

Metastatic clear cell renal cell carcinoma (mccRCC) is a fatal disease with a dismal prognosis and has been proved to be resistant to chemotherapy and radiotherapy. The first-line treatment for mccRCC is still systemic therapy including immunotherapy and targeted therapy (1, 5). While it is common for mccRCC to develop drug resistance during systemic drug therapy (6–9).

To date, effective therapeutic strategies for mccRCC remain absent. So, we should actively explore the treatment of the primary tumor to improve the poor prognosis for patients with mccRCC (1). Radical nephrectomy in patients with metastatic disease, termed cytoreductive nephrectomy (CN), was not the first-line treatment of mccRCC. But, as an alternative treatment for mccRCC, CN has been shown to improve the prognosis and overall survival of some patients with mccRCC (1, 10, 11). Due to individual variation among patients, different patients may derive different clinical benefits from CN. Clear consensus on what kind of mccRCC patients are suitable for CN is still lacking (12–14).

As a consequence, we aimed to identify optimal candidates for CN. To satisfy this need, we used the SEER database to develop a nomogram for predicting suitable candidates for CN in patients with mccRCC.

## Materials and Methods

### Data Source and Study Population

SEER*Stat software (version 8.3.9) was used to extract data from the Surveillance, Epidemiology, and End Results (SEER) database. We had applied for access to the publicly accessible database, so there is no need for another ethical review.

According to the International Classification of Disease for Oncology (ICD-O), we selected patients with renal tumors (ICD-O code64) from the SEER database (2010–2015). Inclusion criteria (1): Patients with distant metastases (AJCC 7th M1) (2); First and only one primary tumor (Renal Cell Carcinoma). Exclusion criteria (1): TNM stage is unknown (2); Survival information is unclear (3); Surgical information is unclear (4); Metastatic status is unclear (5); Non-unilateral tumor. All baseline data, clinical data, and survival data were collected and retrospectively analyzed.

**Figure 1:**
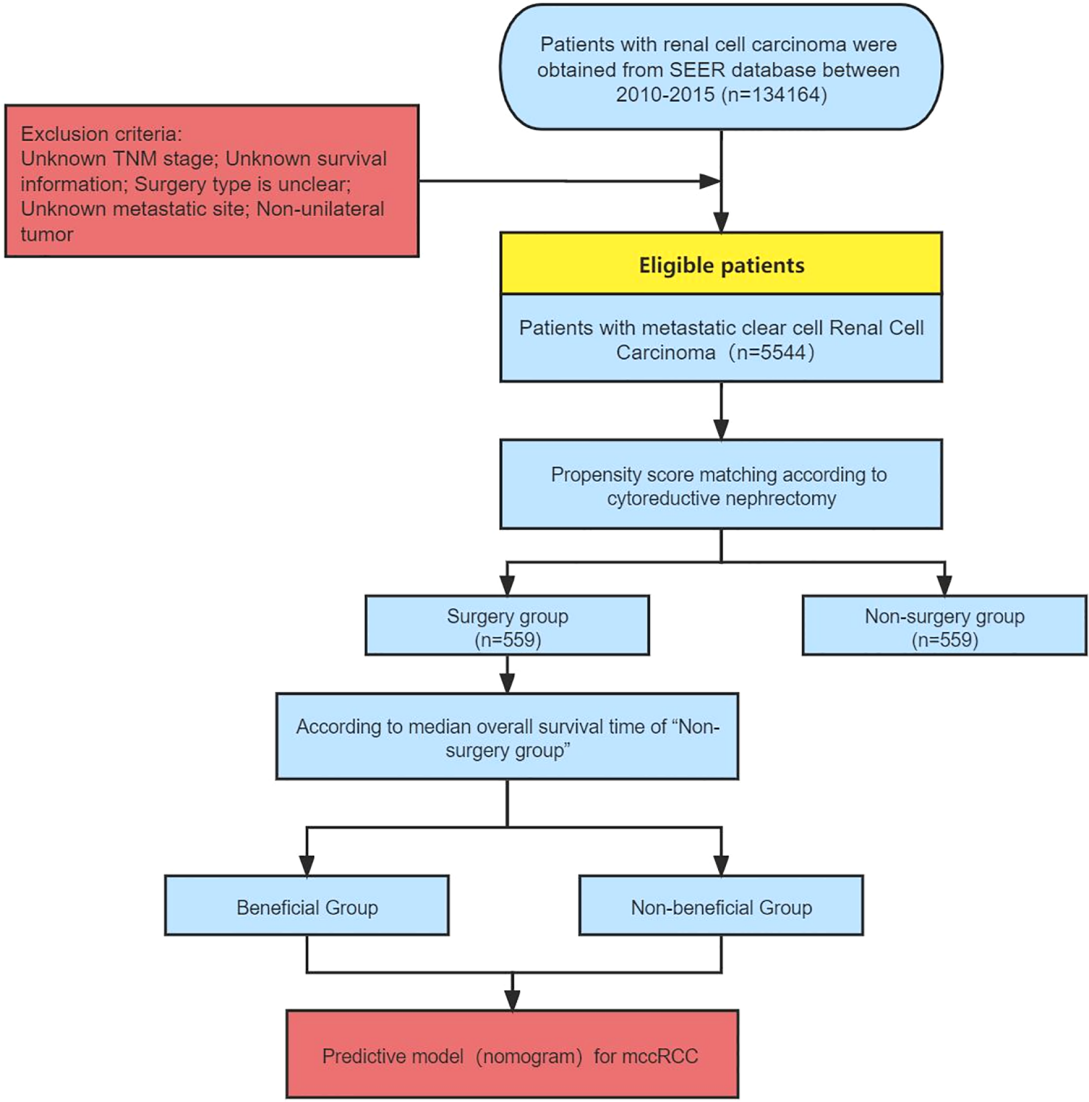
Flowchart of patient selection and establishment of the predictive model.

Surgery (cytoreductive nephrectomy) was defined as radical nephrectomy (surgery code: 50). The selection of the study population and establishment of the predictive model was shown in **Figure 1**.

### Statistical Analysis

These patients were divided into surgery and no-surgery groups by whether or not they underwent CN. Estimated annual percentage changes (EAPC) were quantified to analyze the temporary trend of the treatment type. Overall survival (OS) and cancer-specific survival (CSS) were estimated by using the Kaplan-Meier method and the log-rank test. Multivariate Logistic regression (MLR) models were used to predict the recipients of CN to prove the necessity of propensity scoring matching (PSM). Multivariate Cox regression was used to determine the independent risk factors which would be included in nomogram.

Propensity scoring matching (PSM) was used to minimize potential bias and increase the precision of our research, as clinical decisions may be influenced by baseline characteristics of patients. We included matched covariates that may affect our research (age at diagnosis, gender, race, pathologic grading, TNM stage, whether systemic therapy and radiotherapy were administered, and metastatic status).

These baseline characteristics were 1:1 matched between the two groups using the nearest-neighbor method (caliper was 0.01). After matching, the Chi-square test was used to determine the significance of the difference in categorical variables. The univariate Cox regression was used to compare groups for categorical variables. We calculated the hazard ratio (HR) with a 95% confidence interval (95% CI). Statistical analyses and image drawing were performed with R software version-4.0, SPSS (version 25). and Graph prism 8.0. P-value<0.05 was considered statistically significant.

### Establishment and Validation of the Nomogram

After PSM, we defined patients in the surgery group as “Surgical-benefit” if their survival time exceeded the median OS time of the non-surgery group; we then classified patients in the surgery group as “beneficial group” (survival time >7 months) and “non-beneficial group” (Survival time ≤ 7months).

We developed this prediction model by using multivariate logistic regression analysis to identify patients with mccRCC who may benefit from CN. The matched surgery group (n=559) was randomly divided into two groups for training and validation in a ratio of 7:3. The Logistic regression model comprised the indepent predictor variables from MCR including age, sex, race, pathologic grading, T stage, N stage, systemic treatment, and multiple organ metastases.

This prediction model was based on the training group and was displayed in a nomogram. The probability that mccRCC patients would benefit from CN was calculated by adding the scores for each selected variable. The area under the receiver operating characteristic (AUC) can be used to determine the prediction efficiency (sensitivity and specificity). To compare predicted and observed outcomes, a calibration plot and the Hosmer-Lemeshow test were used (a p-value greater than 0.05 was considered to be a good model fit).

### Clinical Application

A decision curve analysis (DCA) was used to determine the utility of the nomogram in clinical decision-making. We devised a surgical-benefit classification system for patients with mccRCC and categorized them into two groups according to their response to CN (1). Surgery-Benefit group, whose benefit possibility>0.5, indicating that these patients could benefit from CN, which would then lengthen survival time of these patients (2). Surgery-No Benefit group with a probability of benefit ≤ 0.5, indicating that these patients have a tiny chance of benefiting from CN.

As shown by our prediction model, we used Kaplan–Meier survival analysis to compare the OS of the “Surgery-Benefit group” and the “Surgery-No Benefit group” to verify that the model is capable of identifying patients who may benefit from CN and evaluate the clinical utility of the model.

## Result

### Selection of Patients and Baseline Characteristics

**Figure 2:**
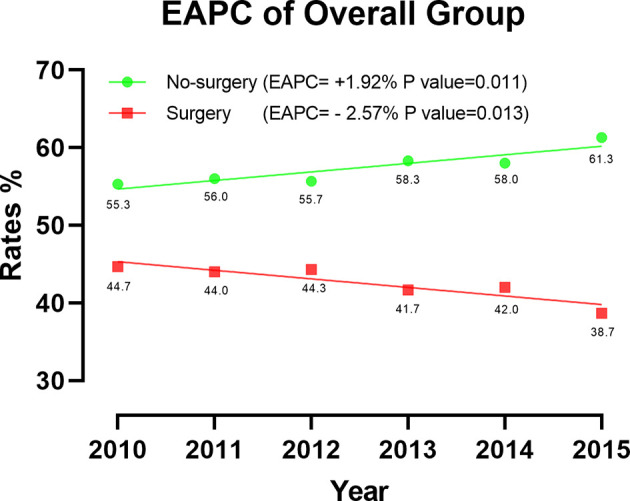
Estimated annual changes (EAPC) of overall patients between 2010-2015.

A total of 5544 patients was included in this research. Among them, 2352 patients (42.4%) underwent CN. The rate of CN recipients decreased over time in 2010 -2015. (EAPC=-2.57%, P=0.013, CI: -3.71% to -1.41%) (**Figure 2**). Multivariate Logistic regression (MLR) of overall patients showed that the independent predictors of CN were age, grade, T stage, and the number of metastatic organs (**Table 1**).

**Table 1:** Multivariable logistic regression models predicting probability of CN recipients.

Variable		Odds Ratio	95% Confidence Interval	*P-*value
**Age**				
	**< 65**	**Ref**	**—**	**—**
	**≥65**	**0.64**	**0.54~0.75**	**<0.001**
**Gender**				
	**Male**	**Ref**	**—**	**—**
	**Female**	**0.95**	**0.80~1.14**	**0.657**
**Laterality**				
	**Left**	**Ref**	**—**	**—**
	**Right**	**0.76**	**0.64~0.89**	**0.004**
**Race**				
	**White**	**Ref**	**—**	**—**
	**Black**	**0.82**	**0.62~1.07**	**0.229**
	**Asian or Pacific Islander**	**1.14**	**0.82~1.59**	**0.514**
	**American Indian/Alaska Native**	**0.96**	**0.44~2.05**	**0.930**
**Grade**				
	**G1**	**Ref**	**—**	**—**
	**G2**	**2.59**	**1.50~4.53**	**0.004**
	**G3**	**3.18**	**1.87~5.49**	**<0.001**
	**G4**	**8.91**	**5.13~15.75**	**<0.001**
	**Gx**	**0.11**	**0.06~0.19**	**<0.001**
**T-stage**				
	**T1**	**Ref**	**—**	**—**
	**T2**	**1.21**	**0.94~1.56**	**0.206**
	**T3**	**6.04**	**4.8~7.64**	**<0.001**
	**T4**	**1.41**	**1.06~1.88**	**0.049**
**N-stage**				
	**N0**	**Ref**	**—**	**—**
	**N1**	**0.28**	**0.24~0.34**	**<0.001**
**Bone**				
	**Yes**	**Ref**		**—**
	**No**	**1.85**	**1.52~2.25**	**<0.001**
**Brain**				
	**Yes**	**Ref**	**—**	**—**
	**No**	**3.34**	**2.52~4.43**	**<0.001**
**Liver**				
	**Yes**	**Ref**	**—**	**—**
	**No**	**2.53**	**2.03~3.16**	**<0.001**
**Lung**				
	**Yes**	**Ref**		**—**
	**No**	**2.57**	**2.16~3.05**	**<0.001**
**Systemic therapy**				
	**Yes**	**Ref**		**—**
	**No/Unknown**	**0.03**	**0.03~0.04**	**<0.001**
**Radiotherapy**				**—**
	**Yes**	**Ref**	**—**	**—**
	**No/Unknown**	**1.37**	**1.10~1.72**	**0.018**

Ref, reference. Red text was regarded as statistical difference.

There were significant differences in age, sex, race, pathologic grading, TNM stage, distant metastasis, radiation therapy, and systemic therapy before matching, which further demonstrated that the baseline characteristics of the 2 groups were unbalanced. After PSM, 559 patients were retained in each group and the baseline characteristics were well-balanced (P>0.1) (**Table 2**).

**Table 2:** Baseline characteristics of the study population.

Variable	Before PSM		*P-*value		After PSM		*P-*value	
	Sugery	Non-sugery			Sugery	Non-sugery		
	n=2352	n=3192			N=559	N=559		
**Age**			**<0.001**				**0.628**
** <65**	**1529 (65.0)**	**1436 (45.0)**		**326 (58.3)**		**317 (56.7)**	
** ≥65**	**823 (35.0)**	**1756 (55.0)**		**233 (41.7)**		**242 (43.3)**	
**Gender**			**<0.001**				**0.486**
** Male**	**1672 (71.1)**	**2134 (66.9)**		**366 (65.5)**		**378 (67.6)**	
** Female**	**680 (28.9)**	**1058 (33.1)**		**193 (34.5)**		**181 (32.4)**	
**Laterality**			**0.078**				**0.675**
** Left**	**1239 (52.7)**	**1604 (50.3)**		**282 (50.4)**		**274 (49.0)**	
** Right**	**1113 (47.3)**	**1588 (49.7)**		**277 (49.6)**		**285 (51.0)**	
**Race**			**<0.001**				**0.632**
** White**	**1958 (83.2)**	**2590 (81.1)**		**440 (78.7)**		**457 (81.8)**	
** Black**	**181 (7.7)**	**372 (11.7)**		**72 (12.9)**		**62 (11.1)**	
** Asian or Pacific Islander**	**184 (7.8)**	**186 (5.8)**		**41 (7.3)**		**34 (6.1)**	
** American Indian/Alaska Native**	**29 (1.2)**	**44 (1.4)**		**6 (1.1)**		**6 (1.1)**	
**Grade**			**<0.001**				**0.406**
** G1**	**29 (1.2)**	**45 (1.4)**		**20 (3.6)**		**13 (2.3)**	
** G2**	**350 (14.9)**	**191 (6.0)**		**103 (18.4)**		**101 (18.1)**	
** G3**	**861 (36.6)**	**285 (8.9)**		**149 (26.7)**		**167 (29.9)**	
** G4**	**821 (34.9)**	**98 (3.1)**		**89 (15.9)**		**74 (13.2)**	
** Gx**	**291 (12.4)**	**2573 (80.6)**		**198 (35.4)**		**204 (36.5)**	
**T-stage**			**<0.001**				**0.343**
** T1**	**236 (10.0)**	**902 (28.3)**		**128 (22.9)**		**119 (21.3)**	
** T2**	**324 (13.8)**	**894 (28.0)**		**136 (24.3)**		**119 (21.3)**	
** T3**	**1533 (65.2)**	**848 (26.6)**		**198 (35.4)**		**226 (40.4)**	
** T4**	**259 (11.0)**	**548 (17.2)**		**97 (17.4)**		**95 (17.0)**	
**N-stage**			**<0.001**				**0.754**
** N0**	**1660 (70.6)**	**1869 (58.6)**		**359 (64.2)**		**365 (65.3)**	
** N1**	**692 (29.4)**	**1323 (41.4)**		**200 (35.8)**		**194 (34.7)**	
**Bone**			**<0.001**				**0.577**
** Yes**	**709 (30.1)**	**1411 (44.2)**		**210 (37.6)**		**200 (35.8)**	
** No**	**1643 (69.9)**	**1781 (55.8)**		**349 (62.4)**		**359 (64.2)**	
**Brain**			**<0.001**				**1.00**
** Yes**	**178 (7.6)**	**472 (14.8)**		**60 (10.7)**		**60 (10.7)**	
** No**	**2174 (92.4)**	**2720 (85.2)**		**499 (89.3)**		**499 (89.3)**	
**Liver**			**<0.001**				**0.809**
** Yes**	**259 (11.0)**	**826 (25.9)**		**90 (16.1)**		**94 (16.8)**	
** No**	**2093 (89.0)**	**2366 (74.1)**		**469 (83.9)**		**465 (83.2)**	
**Lung**			**0.001**				**0.145**
** Yes**	**1415 (60.2)**	**2061 (64.6)**		**314 (56.2)**		**339 (60.6)**	
** No**	**937 (39.8)**	**1131 (35.4)**		**245 (43.8)**		**220 (39.4)**	
**Multiple organ metastasis**			**<0.001**				**0.375**
** None**	**387 (16.5)**	**239 (7.5)**		**76 (13.6)**		**75 (13.4)**	
** Only one**	**1458 (62.0)**	**1564 (49.0)**		**327 (58.5)**		**307 (54.9)**	
** Multiple**	**507 (21.6)**	**1389 (43.5)**		**156 (27.9)**		**177 (31.7)**	
**Systemic therapy**			**<0.001**				**0.830**
** Yes**	**1424 (60.5)**	**224 (7.0)**		**128 (22.9)**		**124 (22.2)**	
** No/Unknown**	**928 (39.5)**	**2968 (93.0)**		**431 (77.1)**		**435 (77.8)**	
**Radiotherapy**			**<0.001**				**0.556**
**Yes**	**614 (26.1)**	**995 (31.2)**		**171 (30.6)**		**161 (28.8)**	
**No/Unknown**	**1738 (73.9)**	**2197 (68.8)**		**388 (69.4)**		**398 (71.2)**	

PSM, propensity score matching. Red text was regarded as statistical difference.

**Figure 3:**
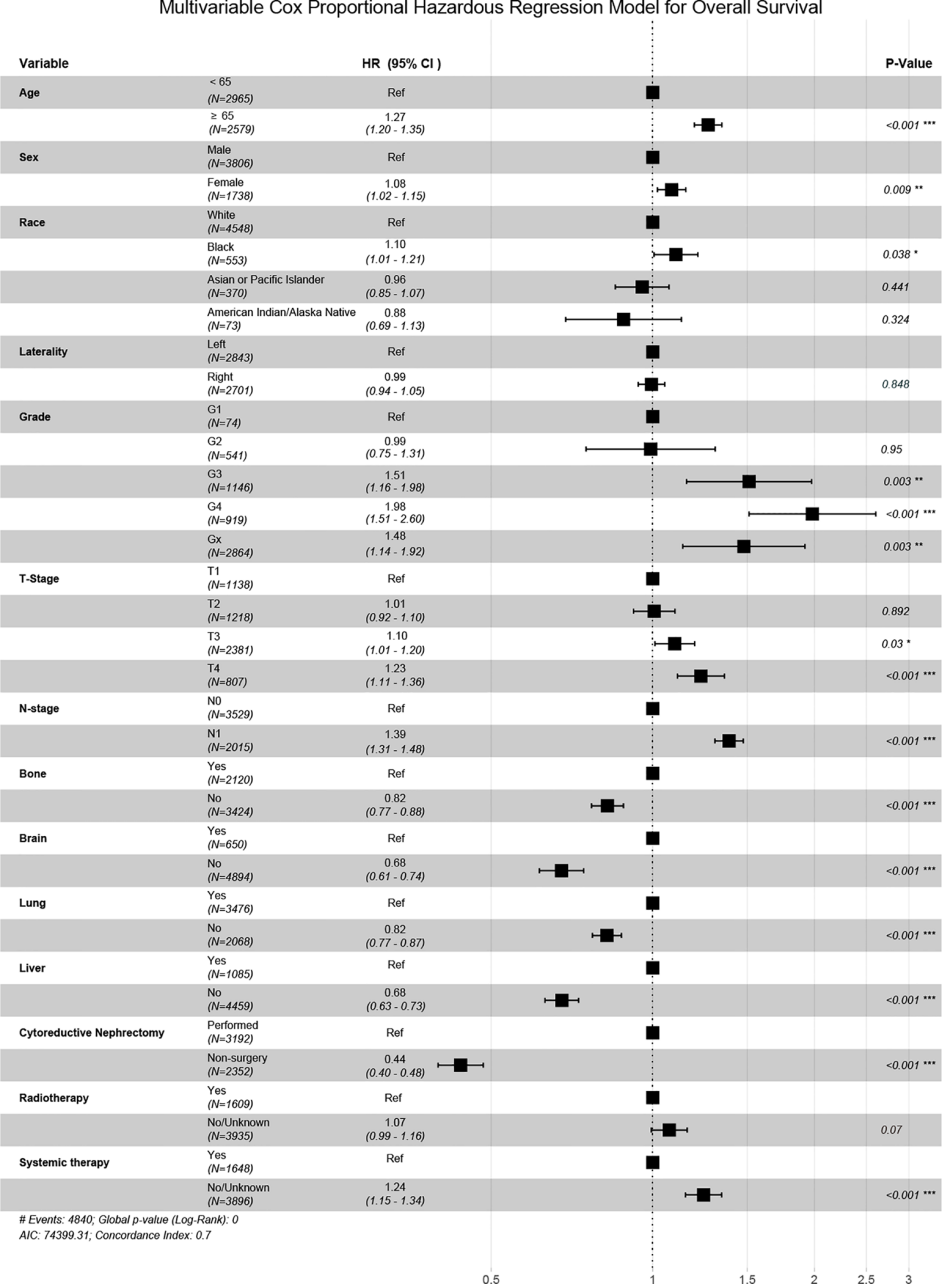
The forest plot of multivariate Cox regression analysis.

The Independent prognostic predictors for OS contained age, gender, race, pathological grade, TNM stage, metastatic site, CN and systemic therapy in our multivariate Cox regression (**Figure 3**). These variables were included in the nomogram.

### The Relationship Between Cytoreductive Nephrectomy and Survival

**Figure 4:**
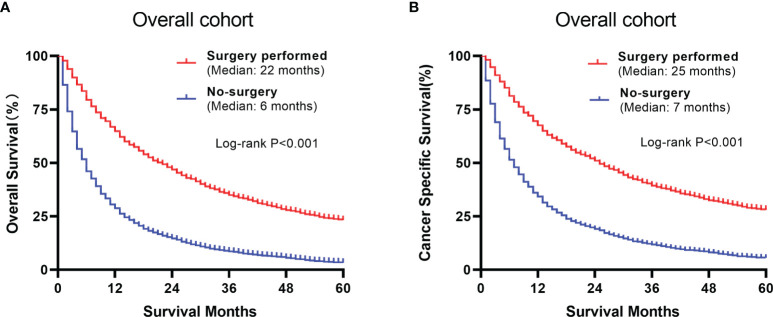
Comparison between the surgery and non-surgery groups of the overall population. **(A)** Overall survival of the 2 groups using Kaplan-Meier analysis. **(B)** Cancer specific survival plots of the overall population.

**Figure 5:**
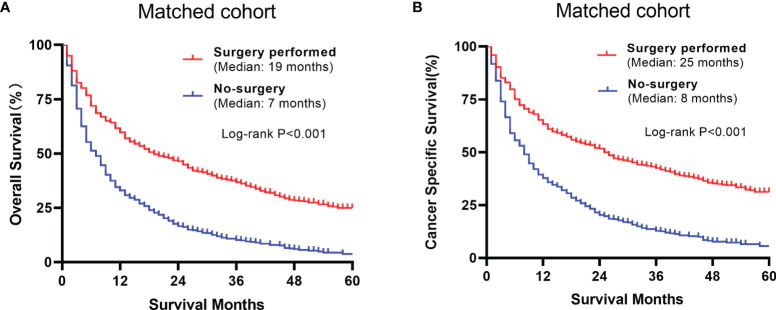
Comparison between the surgery and non-surgery groups of the matched group **(A)** Overall survival of the 2 matched groups **(B)** Cancer specific survival plots of the matched groups.

**Figure 6:**
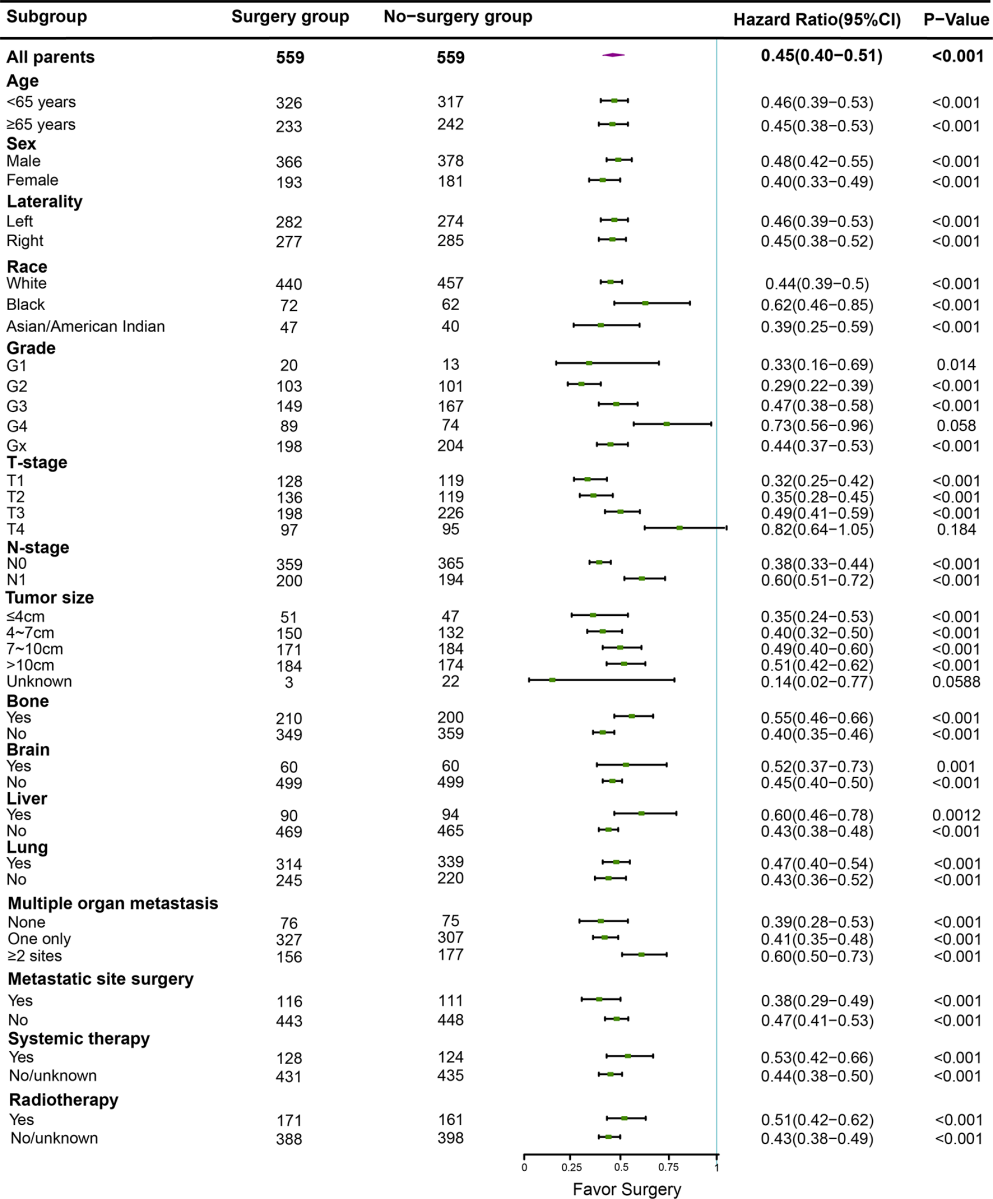
In different subgroups, overal survival was analyzed between the surgery and non-surgery groups, the median dot of each group represents Hazard Ratio (HR), horizontal lines represent 95% confidence interval (95% CI).

The overall and paired cohorts of the two groups were compared and analyzed. There are statistical differences in OS and CSS. In overall cohorts, the median OS and CSS of the no-surgery group are 6 months and 7 months, respectively. For the surgery group, the median OS and CSS are 22 months and 25 months (**Figure 4**). The median OS and CSS of the matched no-surgery group are 7 months and 8 months, whereas the matched surgery group are 19 months and 25 months respectively (**Figure 5**). Additionally, patients who underwent CN gained improved overall survival in most subgroups (**Figure 6**).

### Nomogram to Identify Benefit Candidate for Cytoreductive Nephrectomy

The results above showed that patients who received CN had a significantly longer survival time than those who did not have surgery. Thus, to distinguish the patients who are suitable for CN, we defined patients who survive longer than the median OS of the non-surgery group (7 months) as those who can get benefit from CN.

**Figure 7:**
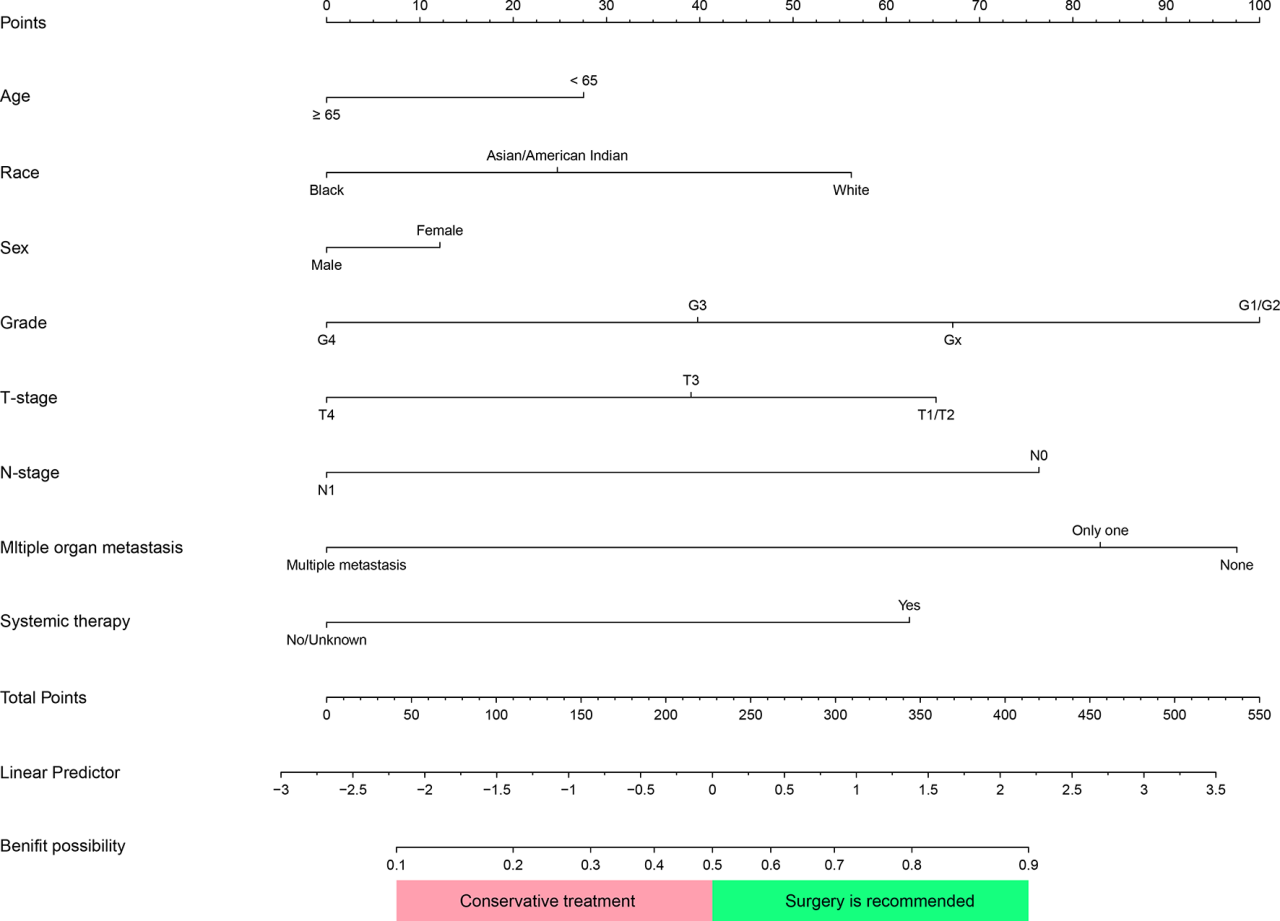
Nomogram was used to identify patients with metastatic clear cell renal cell carcinoma(mccRCC) who would benefit from Cytoreductive Nephrectomy. Corresponding scores of each variable were added to get a total score, then calculating the possibility of getting benefit. Patients whose Benefit possibility>0.5 were recommended for this surgery.

In the surgery group, 367 (65.7%) patients were defined as “Beneficial Group”, their survival time was above 7 months. The remaining were classified as “Non-beneficial Group”. The variables “Age, sex, race, pathologic grading, TNM stage, systemic therapy, and multiple distant metastases” of the nomogram were selected *via* MCR in overall patients. Based on multivariate Logistic regression analysis, we present a predictive model in the form of a nomogram to predict which mccRCC patients in the training group will benefit from CN (**Figure 7**).

**Figure 8:**
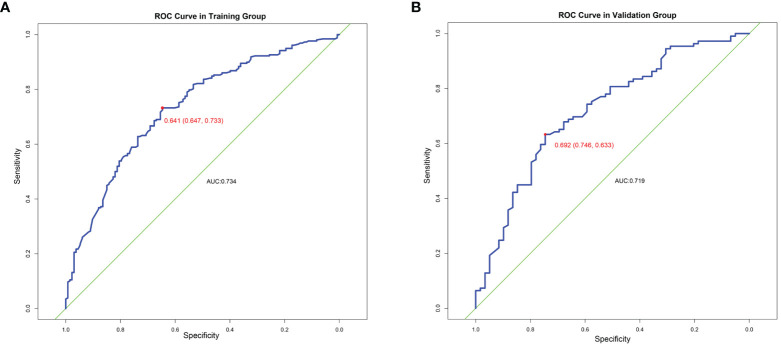
ROC curve on training group **(A)** and validation group **(B)**.

**Figure 9:**
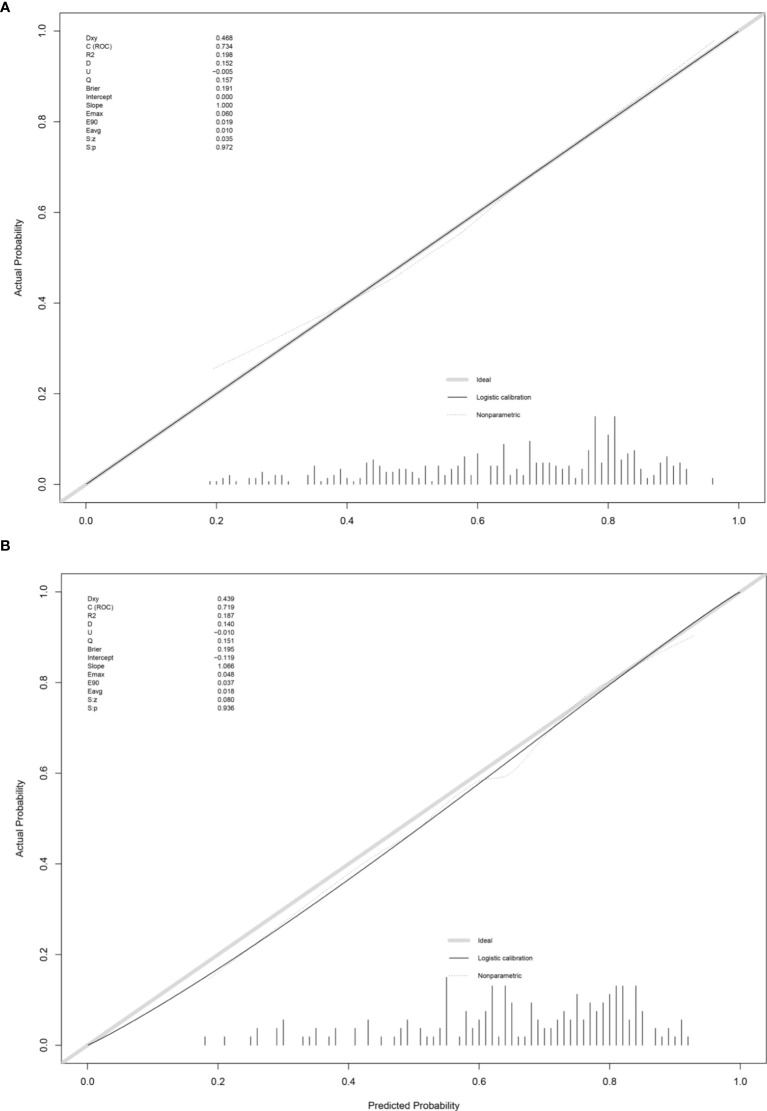
Calibration plot of training group **(A)** and validation group **(B)**.

This prediction model can well identify suitable patients for CN in both the training group (AUC=0.734) and the validation group (AUC=0.71) (**Figure 8**). The actual calibration curve observation results for the training group and validation group are in perfect agreement with the nomogram’s projected outcomes (**Figure 9**).

### Clinical Application of the Nomogram

Superimposing all corresponding scores of each variable in a nomogram to calculate the surgery-benefit probability. Based on the total score, candidates with a predicted probability greater than the 0.5 cutoff point were classified as “surgical benefit candidates”. Otherwise, they are classified as “non-surgical benefit candidates.”

**Figure 10:**
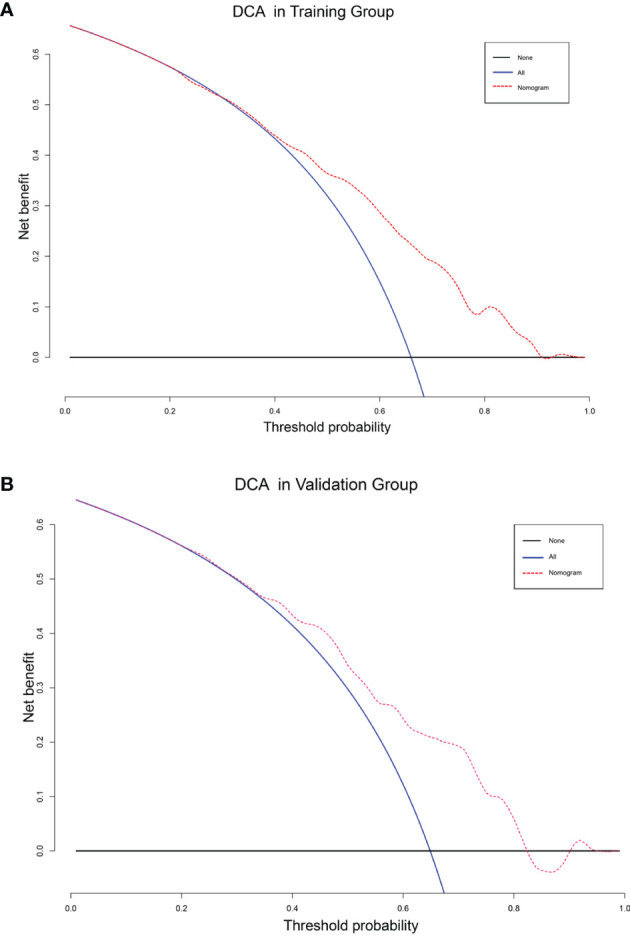
Decision curve analysis of training group **(A)** and validation group **(B)**.

**Figure 11:**
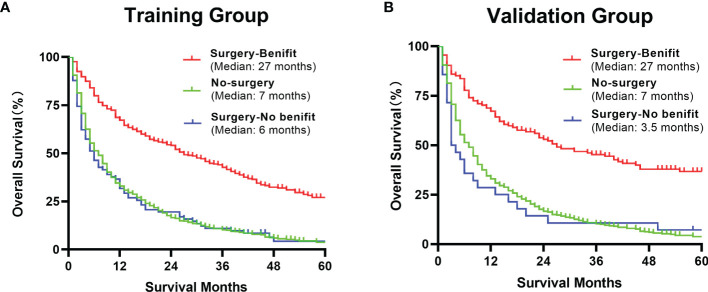
Kaplan–Meier analysis of a comparison of benefit candidates, no-benefit candidates and no-surgery patients in the training group **(A)** and validation group **(B)** after using our nomogram.

The DCA analysis demonstrated the clinical value of the nomogram (**Figure 10**). We used Kaplan-Meier analysis to compare the OS of “Surgery-Benefit group”, “Surgery-No benefit group” and “No-surgery”. In the training and validation groups, the survival of different groups was accurately distinguished, confirming its clinical value (**Figure 11**).

## Discussion

This is the first study based on the SEER database for CN selection in mccRCC patients. We designed and validated a predictive model for identifying probable mccRCC patients who would potentially benefit from CN. In general, prediction efficiency and practical value were acceptable, and the prediction factor of this model was easily acquired, which increased the utility during clinical application.

The results of EAPC indicated a downward trend in mccRCC patients undergoing CN surgery in 2010-2015. Based on the good performance of the surgery, we should make full use of the effectiveness of CN, which further demonstrated the importance of our research.

Our study showed that the majority of mccRCC patients who underwent CN lived longer than those without cytoreductive surgery in matched groups, which is consistent with previous research (1, 15–17). Despite our study revealing that CN can improve survival of mccRCC patients to a certain amount, not all mccRCC patients survived longer than patients without surgery. Additionally, surgery raised the extra physical and economic toll on patients unsuitable for surgery according to our model.

In our nomogram, pathological grade and the number of distant metastases were the strongest predictors that affect surgical outcomes.

Patients with pathological Grade 4 (including sarcomatoid degeneration) had a bad prognosis. Renal tumor puncture biopsy can determine the pathological type and grade of the tumor. Additionally, it provides a clear pathology diagnosis that can be used to guide targeted therapy and immunotherapy (18, 19). For patients with mccRCC who intend to use our predictive model, a biopsy is suggested since the nuclear grading and presence of sarcomatoid degeneration had a significant impact on the surgical benefit.

The condition of metastasis had a significant impact on the efficiency of CN. It is easier to improve survival time with CN in individuals with 0 or 1 major organ metastasis including liver, lung, bone and brain. This may be because the tumor-reduction effect of CN was not as effective in patients with multiple organ metastasis as it was in those with single or no major organ metastasis. After CN, metastatic tumors continue to cause significant injury. There are no reliable studies that demonstrate a link between the metastatic status of patients with mccRCC and the efficiency of CN,

Kaplan-Meier analysis was carried out to explore the relationship between the number of metastatic sites and prognosis in the surgical group before matching, and we found that the effect of CN became worse and worse with the increase of the number of organ metastases (Listed in **Supplementary Figures S1**, **S2**).

As a first-line treatment option for mccRCC, targeted therapy and immunotherapy improve survival conditions for mccRCC patients (1, 15, 20). A noted clinical trial showed that for intermediate-risk or poor-risk metastatic renal-cell carcinoma patients sunitinib alone was not inferior to CN followed by sunitinib (17). Another study also demonstrated that a period of sunitinib therapy before CN improves overall survival compared with immediate CN followed by sunitinib although deferred CN did not improve progression-free rate (21). Our research further demonstrated that CN in combination with systemic therapy can further prolong OS in mccRCC patients. Therefore, patients who have undergone targeted therapy and immunotherapy and are expected to be able to continue treatment following surgery have a greater chance of benefiting from CN.

Renal clear cell carcinoma is not radiotherapy sensitive (1). However, preoperative radiotherapy may increase the chance of surgical benefit in metastatic renal clear cell carcinoma, which may be related to the reduction of tumor burden caused by distant metastasis by radiotherapy (22, 23). However, our data showed that Radiotherapy can not be regarded as an independent predictor. Whether radiation therapy can improve the prognosis of CN will need to be studied in further research.

The model is a useful auxiliary tool to assist in determining which patients are suitable for tumor reduction surgery at the time of diagnosis. In clinical practice, physicians can use our nomogram to determine the value of individual patients who might benefit from CN. Preoperative evaluation data can be easily accessed. Patients classified as surgical benefit are more likely to benefit from CN and have better outcomes. For these patients, surgical treatment, in addition to targeted therapy and immunotherapy, could be an effective treatment option in this case. However, it is not recommended to perform CN on patients classified as non-surgical benefit candidates. Thus, a systematic treatment strategy combining targeted therapy and immunotherapy will be more rational.

However, the definitive effect of CN is still disputed, and there are no clear criteria for selecting individuals with mccRCC who may benefit from surgical treatment. Multiple clinical indicators may be more predictive than a single index in clinical decision-making. A prediction model could be an ideal auxiliary tool in this scenario for selecting the best patients. As a consequence, our research can facilitate doctors, improve treatment for mccRCC patients, and contribute to future research.

While our prediction model is fairly accurate, our research has some limitations. To begin, the SEER database does not include information about the patient’s basic condition or if they suffered complications, which may have a biased effect on the patient’s surgical treatment choice. Second, current prognostic variables for renal cell carcinoma, including ECOG performance status; tumor necrosis status; laboratory results (hemoglobin, LDH, serum calcium), baseline Karnofsky performance score, time from the initial diagnosis to systemic therapy and whether or not they have clinical symptoms, are temporarily unavailable (24–26). For these reasons, we were unable to carry out identification and risk stratification according to Motzer’s criteria. And we can not obtain information about specific systemic treatment, the use of specific drugs from “systemic therapy” and the timing of CN relative to radiotherapy or systemic treatment, which has an impact on the prognosis of mccRCC patients (27, 28). Our prediction model is based on a population retrospective study. Even if the model fits well, it has not been validated by additional external data and is devoid of prospective research. We still require a huge number of samples for prospective studies to confirm the findings in future work.

## Conclusion

Cytoreductive nephrectomy can improve the survival of metastatic clear cell renal cell carcinoma patients. We build a predictive model to select ideal metastatic clear cell renal cell carcinoma candidates. Clinicians could make a more precise treatment strategy for mccRCC patients.

## Data Availability Statement

The original contributions presented in the study are included in the article/**Supplementary Material**. Further inquiries can be directed to the corresponding authors.

## Author Contributions

YZ: research ideas and drafting drafts. JH: statistical analysis. JY: data extraction and manuscript writing. YX, ZC, WS, JH: conception of research. WH, JY, ZZ, QZ: review of the draft. DZ, WX: quality control. All authors contributed to the article and approved the submitted version.

## Conflict of Interest

The authors declare that the research was conducted in the absence of any commercial or financial relationships that could be construed as a potential conflict of interest.

## Publisher’s Note

All claims expressed in this article are solely those of the authors and do not necessarily represent those of their affiliated organizations, or those of the publisher, the editors and the reviewers. Any product that may be evaluated in this article, or claim that may be made by its manufacturer, is not guaranteed or endorsed by the publisher.

## Supplementary Material

Supplementary Figure 1 Venn diagram of metastatic site in overall patients.

Supplementary Figure 2 Kaplan–Meier analysis of the number of metastases in the overall surgery group.
