# Supplementary figures and images for: Selection of Optimal Candidates for Cytoreductive Nephrectomy in Patients with Metastatic Clear Cell Renal Cell Carcinoma: A Predictive Model Based on SEER Database
Source: Front Oncol. 2022 Jan 21;12:814512. doi: 10.3389/fonc.2022.814512 (PMC8814440; doi:10.3389/fonc.2022.814512)

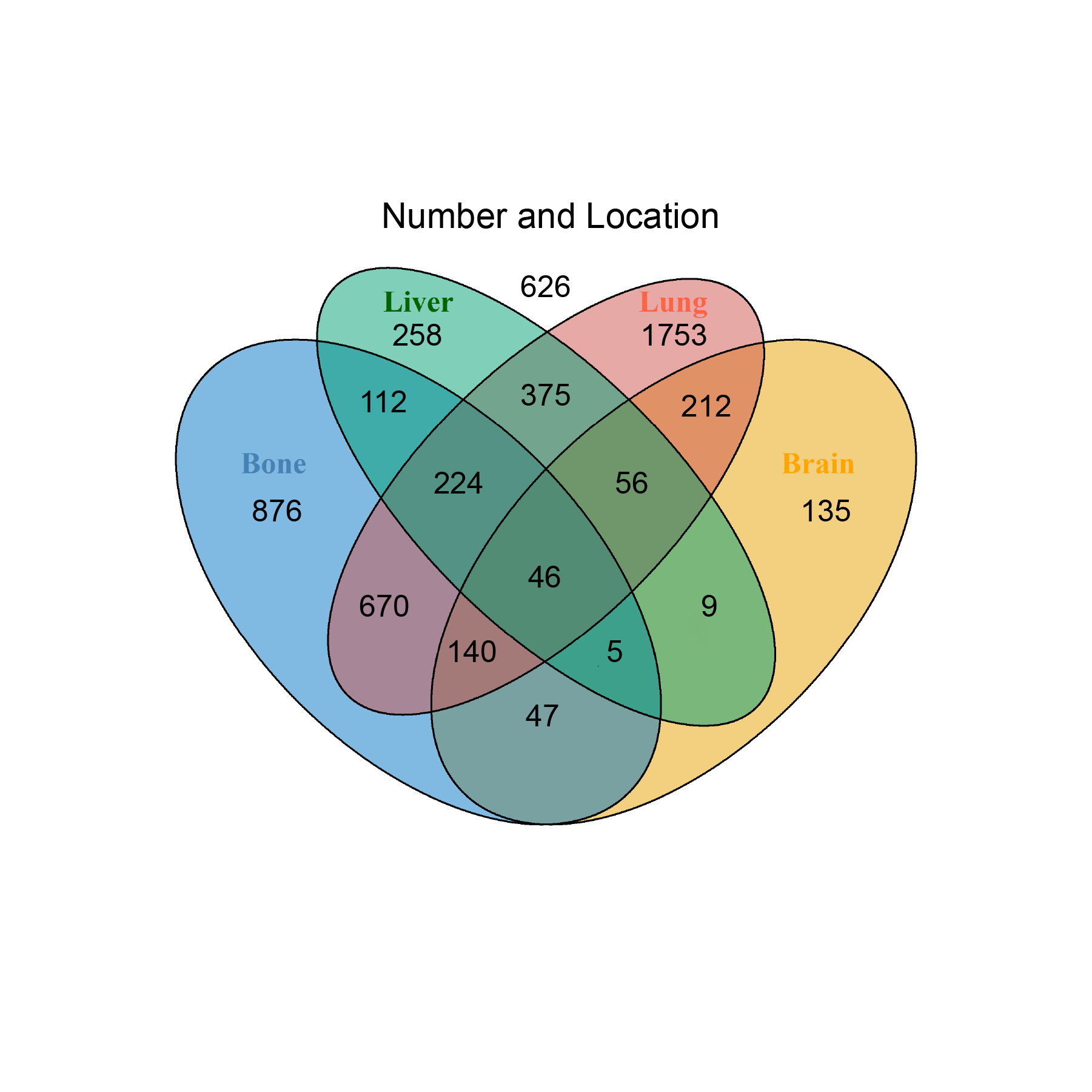

Supplement: Supplementary Figure 1 — Venn diagram of metastatic site in overall patients. [file Image_1.tif]

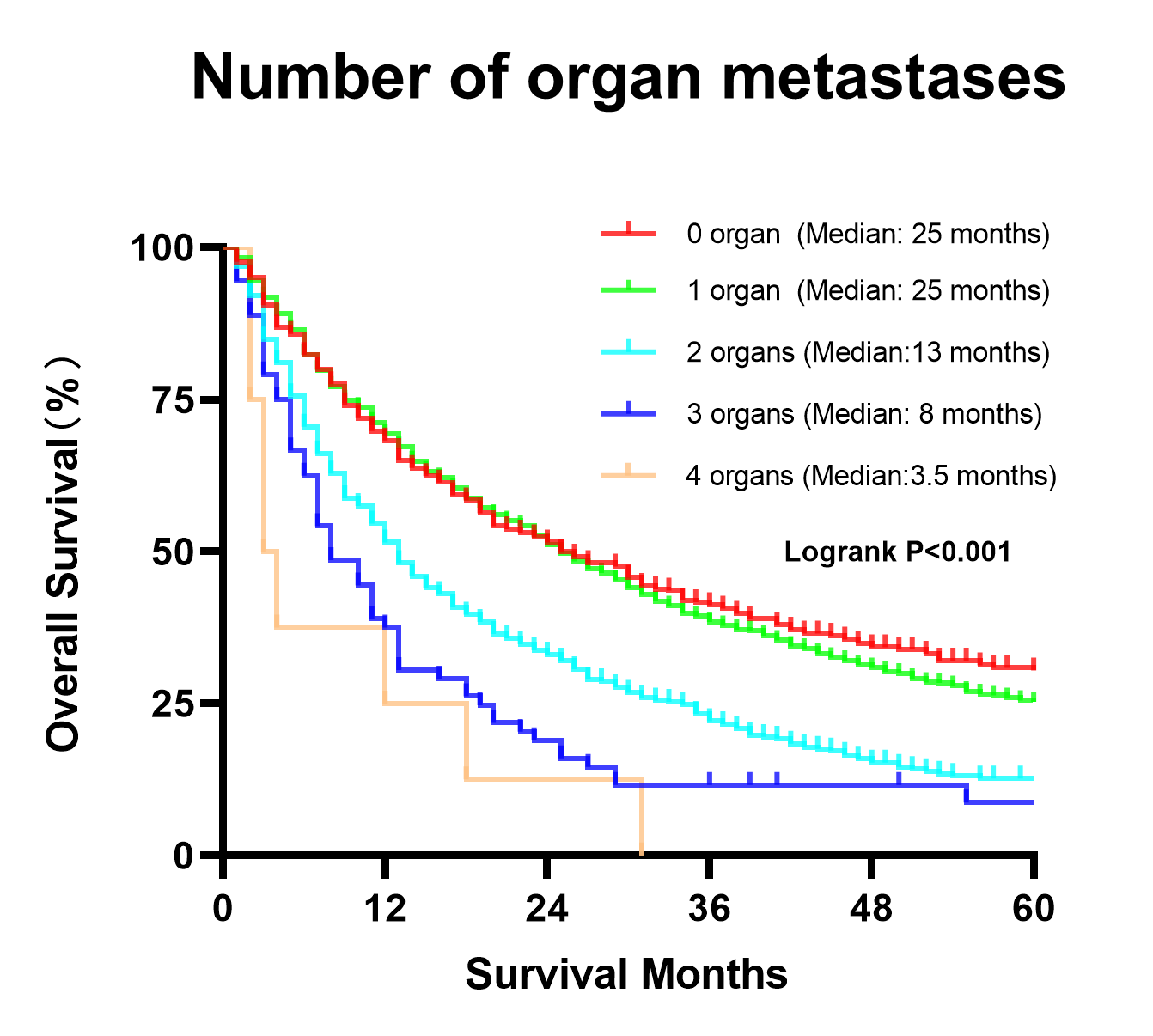

Supplement: Supplementary Figure 2 — Kaplan–Meier analysis of the number of metastases in the overall surgery group. [file Image_2.tif]
